# Micro- and nanoplastics as environmental modifiers of neuroimmune dysfunction in Parkinson’s disease

**DOI:** 10.3389/fnins.2026.1886999

**Published:** 2026-07-01

**Authors:** Sichen Qian, Fei Gao, Peng Wang

**Affiliations:** 1 Zionsville Community High School, Zionsville, IN, United States; 2 Department of Gastroenterology, Shenzhen Hospital of Southern Medical University, Shenzhen, China; 3 Department of Gastroenterology and Hepatology, Indiana University Health, Indianapolis, IN, United States; 4 Brown Center for Immunotherapy, School of Medicine, Indiana University, Indianapolis, IN, United States; 5 Department of Neurosurgery, Heyuan People’s Hospital, Heyuan, China; 6 Department of Neurosurgery, Guangdong Provincial People’s Hospital (Guangdong Academy of Medical Sciences), Southern Medical University, Guangzhou, China

**Keywords:** gut–brain axis, microplastics, nanoplastics, neuroimmunity, neuroinflammation, Parkinson’s disease

## Abstract

Parkinson’s disease (PD) is a progressive neurodegenerative disorder characterized by the loss of dopaminergic neurons and the aggregation of *α*-synuclein, with increasing evidence implicating environmental factors and neuroimmune dysfunction in its pathogenesis. Micro- and nanoplastics (MNPs), ubiquitous environmental pollutants generated from plastic degradation, have recently emerged as potential biological stressors capable of entering the human body and accumulating in sensitive tissues, including the brain. Due to their small size, environmental persistence, and capacity to carry toxic additives and environmental contaminants, these particles can induce oxidative stress, impair mitochondrial and lysosomal function, and activate both innate and adaptive immune responses. This review summarizes current evidence linking microplastic exposure to neuroinflammatory processes relevant to PD, with a particular focus on microglial activation, astrocyte reactivity, peripheral immune involvement, and dysfunction of the gut–brain axis. Although a direct causal relationship between MNPs and PD has yet to be established, and direct human epidemiological evidence linking MNP exposure to PD is currently absent, the immunotoxic and neuroinflammatory effects of these particles suggest that they may contribute to disease susceptibility and progression. Elucidating the interactions between MNPs and neuroimmune pathways may help refine current frameworks linking environmental exposure, neuroimmune dysfunction, and PD susceptibility.

## 1 Introduction

Parkinson’s disease (PD) is a progressive neurodegenerative disorder characterized by the degeneration of dopaminergic neurons in the substantia nigra (Karlsen et al., 1999). It is the second most common neurodegenerative disease, affecting approximately 0.5–1% of individuals aged 65–69 and up to 1–3% of those over 80, with prevalence projected to increase substantially in the coming decades (Kouli et al., 2018). PD is widely recognized as a multifactorial disease, with contributions from genetic predisposition, environmental toxins, and chronic inflammatory processes (Karlsen et al., 1999). Despite extensive research, the precise mechanisms driving disease onset and progression remain incompletely understood.

Micro- and nanoplastics (MNPs) have attracted increasing attention as pervasive and persistent pollutants with potential biological effects. These tiny plastic particles, generated through the degradation of larger plastics, have been detected in drinking water, various food products, and marine organisms (Principi et al., 2025). Owing to their widespread presence and resistance to degradation, MNPs can accumulate in living organisms, raising questions about their long-term biological effects. While the ecological consequences of MNPs are increasingly documented, their potential role in human neurodegenerative diseases remains poorly understood (Liu et al., 2024).

Neuroimmune dysregulation has emerged as a central driver of PD pathogenesis (Tansey et al., 2022). Interactions among microglia, astrocytes, peripheral immune cells, and the gut–brain axis contribute to a self-amplifying cycle of inflammation and neurodegeneration (Muller and Di Benedetto, 2025). *α*-Synuclein, a pathological hallmark of PD, also acts as an immunogenic mediator that links peripheral and central immune responses (Bellini et al., 2025). The gut–brain axis, in particular, may serve as an early site of disease initiation, where environmental exposures and microbial dysbiosis trigger *α*-synuclein aggregation in the enteric nervous system, with subsequent propagation to the brain via neural and immune pathways (Lei et al., 2021; Chao et al., 2020). Given these converging lines of evidence, MNPs represent a hypothetically plausible environmental factor capable of modulating neuroimmune processes relevant to PD. However, their role within this framework remains insufficiently defined (Lin et al., 2026). Therefore, it is important to clarify the evidentiary basis for this hypothesis. Direct experimental evidence linking MNP exposure to PD-relevant neuroimmune mechanisms remains limited and derives primarily from *in vitro* or animal models using pristine nanoparticles at concentrations that may not reflect environmental exposure conditions.

In this review, we examine the intersection between MNP exposure and neuroimmunity in PD. Specifically, we focus on the mechanisms by which MNPs may trigger or exacerbate neuroinflammatory pathways—such as glial activation, peripheral immune dysregulation, and gut–brain axis dysfunction—thereby contributing to the neurodegenerative processes characteristic of the disease. It is important to note that no human epidemiological studies have directly examined associations between MNP exposure and PD risk, and direct evidence for MNP accumulation in human brain tissue remains limited. This review synthesizes current evidence to clarify how plastic pollution may contribute to PD and identifies key knowledge gaps to guide future mechanistic and translational research.

Literature Search Strategy: A literature search was conducted using PubMed, Web of Science, and Scopus databases up to March 2026. Search terms included combinations of “Parkinson’s disease,” “microplastics,” “nanoplastics,” “neuroinflammation,” “neuroimmunity,” “gut–brain axis,” “microglia,” “astrocytes,” and “*α*-synuclein.” Search term combinations included: (“Parkinson’s disease” OR “PD”) AND (“microplastics” OR “nanoplastics” OR “plastic particles”); (“microplastics” OR “nanoplastics”) AND (“neuroinflammation” OR “neuroimmunity” OR “microglia” OR “astrocytes”); (“microplastics” OR “nanoplastics”) AND (“gut-brain axis” OR “gut dysbiosis”); and (“microplastics” OR “nanoplastics”) AND (“*α*-synuclein” OR “alpha-synuclein”). Priority was given to original experimental studies, mechanistic investigations, and recent reviews relevant to neuroimmune and neurotoxic effects of micro- and nanoplastics. Additional references were identified through manual screening of cited literature.

## 2 Neuroimmune mechanisms in PD

Neuroimmune dysregulation in PD operates across central, peripheral, and enteric compartments. While *α*-synuclein aggregation and progressive dopaminergic neuron loss constitute the core pathological features of PD, neuroimmune dysregulation has emerged as an important modifying factor that can amplify these processes. The following sections outline the interrelated immune mechanisms that interact with primary neurodegenerative pathways.

### 2.1 *α*-Synuclein as an immunogenic driver

Aggregated α-synuclein acts as a key immunogenic trigger, engaging microglial Toll-like receptors (TLRs) and activating the NLRP3 inflammasome pathway, culminating in neuroinflammation and dopaminergic neuron loss (Li Y. et al., 2021). NLRP3 activation is further amplified by mitochondrial dysfunction, highlighting this pathway as a potential target for disease-modifying interventions (Liang J. Y. et al., 2025). In addition, chronic neuroinflammation promotes an autoimmune component: stressed dopaminergic neurons upregulate MHC Class I molecules, presenting antigens such as *α*-synuclein or mitochondrial peptides to cytotoxic CD8^+^ T cells (Hobson and Sulzer, 2022). Together, these events establish a feed-forward loop of neuronal stress, immune activation, and progressive neurodegeneration.

### 2.2 Microglia and astrocytes

Microglia, the resident macrophages of the central nervous system, play a key role in regulating neuroinflammation and maintaining homeostasis. Under physiological conditions, microglia are homeostatic. Pathological stimuli, such as misfolded *α*-synuclein aggregates, polarize microglia into a M1 pro-inflammatory and neurotoxic phenotype, also referred to as Disease-Associated Microglia (DAM) (Deczkowska et al., 2018). Recent studies have moved beyond traditional M1/M2 classifications to reveal the heterogeneity of microglial states in PD (Gao et al., 2023). M1-like microglia release cytotoxic cytokines and reactive oxygen species, promote Th1 and Th17 differentiation, and contribute to oxidative stress, mitophagy/autophagy dysfunction, and sustained *α*-synuclein accumulation (Su and Zhou, 2021; Isik et al., 2023; Badanjak et al., 2021; Guo et al., 2022). Chronic M1 activation of microglia thus establishes a self-perpetuating cycle of neuroinflammation and dopaminergic neuron death, and contributes to the progression of neurodegenerative diseases, particularly PD (Gao et al., 2023).

Multiple studies have now revealed that astrocytes can take up α-synuclein and have a role in the removal and degradation of α-synuclein, but in PD, this clearance system becomes overwhelmed, leading to accumulation (Booth et al., 2017). Activated microglia convert astrocytes into a neurotoxic A1 phenotype (neuroinflammatory reactive astrocytes), which triggers neuroinflammation and neuronal loss. The GLP-1 receptor (GLP-1R) agonist NLY01 blocks this microglia-mediated conversion, highlighting the therapeutic potential of targeting glial crosstalk (Kam et al., 2020; Yun et al., 2018). Cytokines, extracellular vesicles, and gap junctions further mediate complex interactions among microglia, astrocytes, and oligodendrocytes, underscoring glial communication as a key axis in PD neurodegeneration (Wang et al., 2025).

### 2.3 Peripheral adaptive immunity

Adaptive immunity contributes significantly to PD pathogenesis. Both CD4^+^ and CD8^+^ T cells infiltrate the brain, where they amplify neuroinflammation and neuronal injury. Among CD4^+^ subsets, Th1 and Th17 cells promote inflammation, whereas regulatory T cells (Tregs) restrain excessive responses; an imbalance favoring Th1/Th17 over Tregs is consistently observed in patients and animal models (Su and Zhou, 2021; DeMaio et al., 2022; Li W. et al., 2021; Sun et al., 2024). *α*-synuclein drives this shift by upregulating ROR*γ*t, enhancing Th17 differentiation, and impairing Treg stability and function (Li et al., 2023). External factors, such as periodontitis and gut colonization by *Veillonella parvula* or *Streptococcus mutans*, exacerbate Th1 infiltration into the central nervous system (CNS) and peripheral tissue (Bai et al., 2023).

CD8^+^ T cells in PD exhibit elevated cytotoxic and differentiation profiles, including expansion of terminally differentiated effector memory (TEMRA) and tissue-resident memory (Trm) subsets, with enhanced expression of granzymes and interferon-γ, infiltrating the substantia nigra even before substantial *α*-synuclein aggregation (Capelle et al., 2023; de Fabregues et al., 2023).

Innate immune cells such as natural killer (NK) cells and monocytes are also dysregulated: NK cells can degrade *α*-synuclein but may release pro-inflammatory cytokines, whereas circulating monocytes adopt a primed pro-inflammatory state, amplifying peripheral immune involvement (Daneshjou et al., 2025; Earls and Lee, 2020; Zhang et al., 2022; Grozdanov et al., 2014). These findings highlight multiple layers of systemic immune dysregulation as contributors to PD progression and therapeutic targets.

### 2.4 Gut–brain axis in PD

The enteric nervous system (ENS) is implicated early in PD pathogenesis, with alterations detectable before central nervous system involvement (Montanari et al., 2023; McQuade et al., 2021). Gut dysbiosis and environmental toxins may initiate *α*-synuclein aggregation in the ENS, which propagates to the brain via the vagus nerve in a prion-like manner (Santos et al., 2019; Zhu et al., 2022).

*α*-synuclein functions as a mechanistic bridge linking gut-derived immune activation to central neurodegeneration, providing a conceptual framework that connects peripheral environmental exposure with CNS pathology (Chen and Mor, 2023; Schaeffer et al., 2020). Gut-derived α-synuclein acts as a peripheral immune modulator, promoting Th17 differentiation and impairing Treg function through RORγt transcription, thereby reinforcing systemic and central neuroinflammation (Li et al., 2023; Park et al., 2023).

Preliminary evidence from animal studies suggests that MNP-induced gut dysbiosis could potentially exacerbate these processes by promoting intestinal barrier disruption and immune activation. However, direct evidence linking MNP exposure to α-synuclein pathology in the enteric nervous system or to PD progression is currently absent. A single study has reported increased intestinal permeability following polystyrene microplastic exposure in mice, and separate studies have demonstrated that gut dysbiosis can promote α-synuclein aggregation in PD models. The integration of these observations suggests that MNPs might influence α-synuclein pathology via gut dysbiosis, but this remains hypothetical and requires direct experimental testing (Lin et al., 2026; Perez-Pardo et al., 2017).

## 3 MNPs: properties, exposure, and biological fate

MNPs comprise a heterogeneous continuum of synthetic polymer particles that are now pervasive across environmental and biological systems. Their physicochemical properties (including size, polymer composition, surface charge, and morphology) govern environmental persistence, transport behavior, and biological interactions. Increasing evidence indicates that the biological effects of MNPs are strongly size-dependent, with MPs primarily contributing to environmental exposure, whereas NPs represent a more biologically active fraction capable of interacting with cellular and subcellular processes (Yang Z. et al., 2023).

### 3.1 Size-dependent properties: MPs versus NPs

MPs are generally defined as plastic particles smaller than 5 mm and originate from the fragmentation of larger plastic materials or from primary sources such as cosmetics and industrial products. Due to their abundance and environmental persistence, MPs constitute the dominant form of plastic exposure in humans and ecosystems. They act as long-lived reservoirs for co-contaminants, including heavy metals, persistent organic pollutants, antibiotic resistance genes, and microbial pathogens, thereby facilitating environmental transport and bioaccumulation (Hu et al., 2022).

NPs, typically ranging from approximately 1 nm to 1 μm, often arise from the progressive degradation of MPs (Lai et al., 2022). Compared with MPs, NPs exhibit markedly distinct physicochemical and biological characteristics. Their small size confers increased mobility and diffusivity, while their high surface area-to-volume ratio enhances adsorption of biomolecules and environmental contaminants (Chen et al., 2023). In biological fluids, NPs readily acquire a dynamic protein corona, which alters their biological identity, cellular uptake, biodistribution, and toxicity (Grumelot et al., 2024). These properties enable NPs to cross biological barriers and interact directly with intracellular structures. Experimental evidence indicates that NPs can penetrate cellular membranes, accumulate in organelles such as mitochondria and lysosomes, and induce oxidative stress, inflammation, and organelle dysfunction.

### 3.2 Human exposure routes and systemic distribution

Human exposure to MNPs occurs predominantly through ingestion and inhalation, with dermal exposure considered less significant (Jahedi and Jaafarzadeh Haghighi Fard, 2025). MPs are widely present in food, drinking water, and airborne particulate matter, representing a continuous source of exposure. Following ingestion, a fraction of these particles undergoes further fragmentation, contributing to the generation of NPs within biological systems (Mortensen et al., 2021).

Particle size determines systemic distribution. While larger MPs are generally confined to the gastrointestinal tract or cleared through excretion, smaller particles—particularly those in the submicron and nanoscale range—can translocate across epithelial barriers. NPs have been shown to cross the intestinal epithelium, enter the circulation, and distribute to secondary organs (Zhou et al., 2025). Studies using pristine polystyrene nanoparticles at concentrations ranging from 0.1–10 mg/kg (oral) or 10–50 μg/mL (*in vitro*) have reported that nanoscale particles can reach the brain within hours after oral exposure in rodent models, with blood–brain barrier (BBB) penetration influenced by surface properties and protein corona composition (Kopatz et al., 2023).

In addition to gastrointestinal uptake, inhaled MNPs can deposit in the respiratory tract, where smaller particles may translocate into systemic circulation. Once internalized, MNPs may accumulate in organs including the liver, spleen, and brain, raising concerns regarding chronic low-dose exposure and long-term biological effects (Lee et al., 2025; Ali et al., 2024).

### 3.3 Tissue distribution, brain accumulation, and biological persistence

The ability of NPs to reach and persist in neural tissue represents a key feature linking environmental plastic exposure to neurobiological outcomes. Following systemic distribution, NPs can cross the BBB and accumulate within the central nervous system. In the brain, these particles may act as persistent sources of oxidative stress and immune activation, interacting with neurons and glial cells (Medley et al., 2023; Anifowoshe et al., 2025).

At the cellular level, NPs can localize to mitochondria and lysosomes, where they disrupt energy metabolism, impair autophagic flux, and promote reactive oxygen species (ROS) generation (Lai et al., 2022; Jahedi and Jaafarzadeh Haghighi Fard, 2025). These processes are closely associated with pathways implicated in neurodegenerative diseases, including *α*-synuclein misfolding and aggregation. Importantly, NP-induced impairment of autophagic digestion may compromise the clearance of aberrant protein aggregates, including α-synuclein, thereby promoting its intracellular accumulation, a core pathological feature of PD (Park et al., 2020).

MPs are less likely to directly access neural tissue due to size constraints. However, they may indirectly contribute to systemic and neuroinflammatory processes by acting as continuous sources of smaller particles and associated toxicants. Therefore, while MPs represent the primary environmental reservoir of exposure, NPs constitute the fraction most capable of penetrating biological systems and eliciting cellular responses (Lin et al., 2026; Lai et al., 2022; Jahedi and Jaafarzadeh Haghighi Fard, 2025; Fang et al., 2025; Yee et al., 2021; Casella and Ballaz, 2024). This distinction helps explain how plastic particles may contribute to neuroimmune dysregulation and neurodegenerative diseases such as PD.

## 4 Immunotoxic effects of MNPs

**Figure 1:**
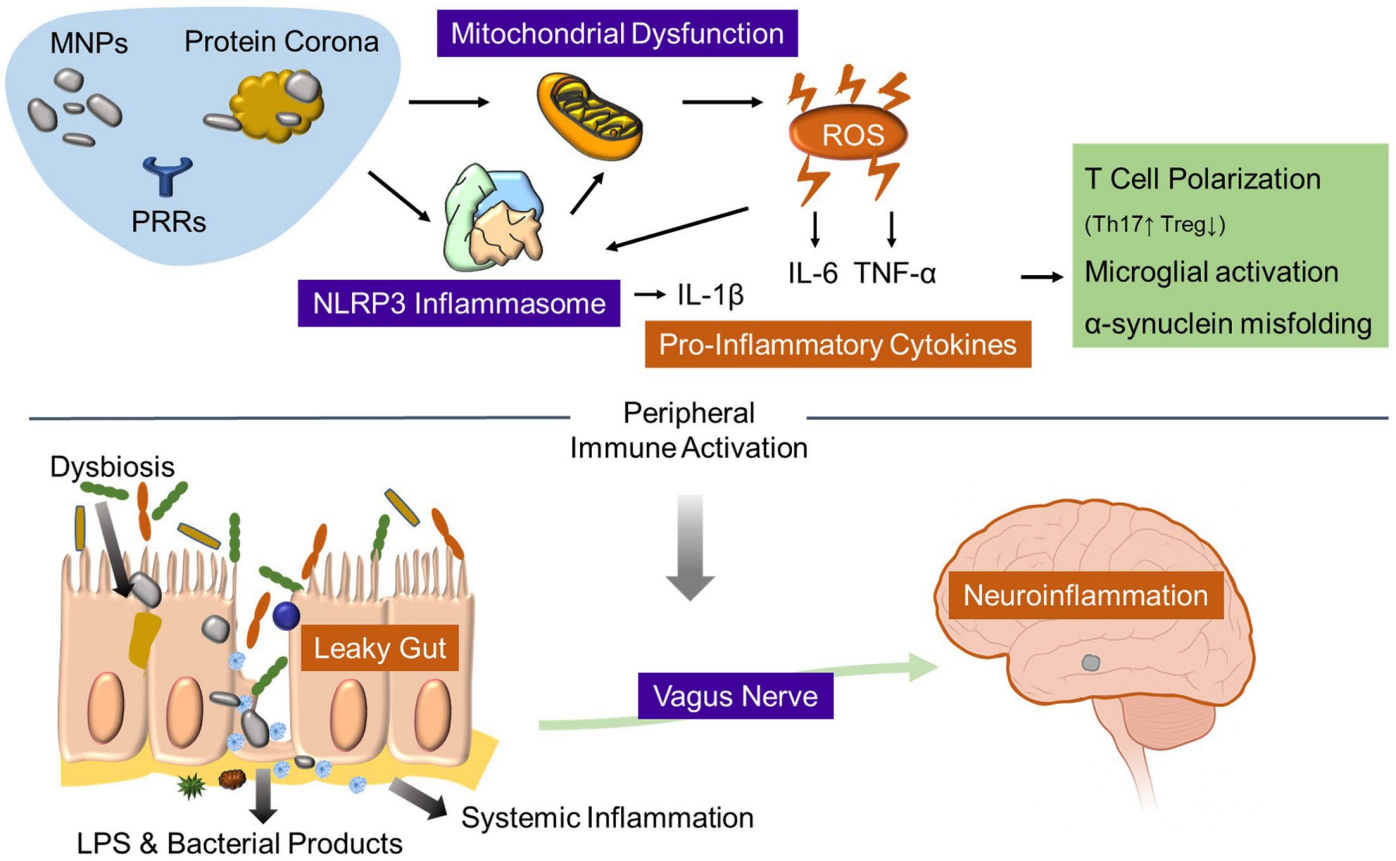
Immunotoxic cascade of micro- and nanoplastics in Parkinson’s disease. Micro- and nanoplastics (MNPs) enter the body primarily via ingestion and inhalation, disrupting gut microbiota and intestinal barrier integrity. Resultant dysbiosis promotes Th17 polarization and Treg suppression, while enteric *α*-synuclein aggregation propagate to the central nervous system via the vagus nerve. At the cellular level, MNPs are recognized by pattern recognition receptors (PRRs) and internalized by innate immune cells, where they induce lysosomal destabilization, mitochondrial dysfunction, and reactive oxygen species (ROS) production, leading to NLRP3 inflammasome activation and pro-inflammatory cytokine release. In parallel, oxidative stress and inflammation promote α-synuclein misfolding and aggregation, establishing feed-forward loops that amplify neuroinflammation and dopaminergic neuron loss.

MNPs are increasingly recognized as immunotoxic agents capable of disrupting both innate and adaptive immune responses. Their small size, high surface area, and capacity to adsorb environmental pollutants and plastic additives enable them to interact with immune cells. These properties may drive oxidative stress and amplify chronic inflammatory signaling (Yang et al., 2022; Leslie et al., 2022). These effects are particularly relevant in PD, where sustained immune activation and neuroinflammation contribute to progressive neurodegeneration. The major immunotoxic mechanisms induced by MNPs are summarized in Figure 1.

### 4.1 Innate immune activation

Innate immune responses represent the first line of defense against particulate exposure and are central to MNP-induced immunotoxicity. The interaction between MNPs and innate immune cells is initiated by particle recognition and uptake. Pattern recognition receptors (PRRs), including Toll-like receptors (TLRs) and scavenger receptors, are thought to mediate the sensing and internalization of plastic particles. In biological environments, the formation of a protein corona further modifies particle identity and influences immune recognition, cellular uptake, and downstream signaling (Panico et al., 2022; Antunes et al., 2023).

Following internalization, nanoscale plastic particles can induce cellular stress responses through multiple interconnected pathways. A key mechanism involves lysosomal destabilization. Particle accumulation within endolysosomal compartments damages the membrane and releases cathepsins into the cytosol, which in turn triggers NLRP3 inflammasome activation. Activation of the NLRP3 inflammasome represents an important downstream event linking MNP exposure to neuroinflammation. Given the established role of NLRP3 signaling in microglia-driven dopaminergic neuron loss in PD (Nguyen et al., 2022; Koprich et al., 2008), nanoparticle-induced inflammasome activation provides a mechanistic bridge between environmental plastic exposure and neurodegenerative vulnerability.

Concurrently, mitochondrial dysfunction and excessive reactive oxygen species (ROS) production activate redox-sensitive pathways such as NF-κB and MAPK signaling (Nguyen et al., 2022; Chang and Chen, 2020). This leads to the release of pro-inflammatory cytokines such as IL-1β, IL-6, and TNF-*α* (Koprich et al., 2008), a neurotoxic milieu that can exacerbate microglial activation and α-synuclein aggregation, which are both central to PD pathogenesis.

Importantly, α-synuclein itself differentially affects innate immune subsets. In monocytes, it enhances migration and inflammatory responses, whereas in macrophages it impairs autophagy and promotes exaggerated cytokine release (Schonhoff et al., 2023; Limanaqi et al., 2024). MNPs may synergize with *α*-synuclein–mediated immune dysfunction and amplify sterile inflammation.

### 4.2 Adaptive immune dysregulation

Beyond innate immunity, MNP exposure can disrupt adaptive immune balance, contributing to systemic immune dysregulation. Studies indicate that nanoscale plastic particles skew T-cell differentiation toward pro-inflammatory phenotypes, particularly Th17 and Th2 responses, while suppressing regulatory T-cell (Treg) function. This shift compromises immune tolerance and promotes chronic inflammation (Yang Q. et al., 2023).

Such immune imbalance is central to PD, where increased Th17 activity and impaired Treg function are hallmarks of disease-associated immune dysfunction. By lowering the threshold for aberrant immune activation, MNP-driven adaptive dysregulation may facilitate autoimmune-like responses against neuronal antigens, including α-synuclein (Ali et al., 2024; Cheng et al., 2024). In addition, MNP exposure has been associated with alterations in cytotoxic CD8^+^ T-cell responses and peripheral immune priming, which may further amplify neuroinflammatory and neuronal injury (Greenland et al., 2025; Moquin-Beaudry et al., 2025).

### 4.3 Gut microbiota–immune interactions

Gut dysbiosis is a recognized feature of PD and may precede motor symptoms (Huang et al., 2021; Huang et al., 2023). It is characterized by depletion of short-chain fatty acid–producing anti-inflammatory bacteria and enrichment of pro-inflammatory taxa (Huang et al., 2023). This imbalance promotes intestinal barrier disruption, systemic inflammation, α-synuclein aggregation, and altered levodopa metabolism (Berthouzoz et al., 2023). Mechanistically, gut dysbiosis promotes increased intestinal permeability (“leaky gut”), facilitating the translocation of microbial products such as lipopolysaccharide (LPS) into systemic circulation (Di Vincenzo et al., 2024; Zhao et al., 2025). This systemic inflammatory state primes peripheral immune cells and enhances their capacity to infiltrate the central nervous system or modulate microglial activation. In parallel, dysbiosis-induced α-synuclein aggregation in the enteric nervous system may propagate to the brain via the vagus nerve, thereby linking peripheral immune activation with central neurodegeneration (Lei et al., 2021; Chen et al., 2019; Cheng and Chiang, 2025).

Intestinal barrier integrity is compromised in PD, evidenced by reduced colonic expression of tight junction proteins such as ZO-1 (van IJzendoorn and Derkinderen, 2019). Decreased expression of microbial metabolite receptors (FFAR2/FFAR3) further supports impaired host–microbiota signaling (Liao et al., 2024).

MNP exposure may exacerbate these processes at multiple levels. Ingestion of MNPs can alter gut microbial composition, induce oxidative stress, and promote pro-inflammatory immune polarization, often skewing toward Th17- and Th2-dominant responses while suppressing Treg activity (Yang et al., 2022; Li et al., 2022). Polyethylene terephthalate and polystyrene particles stimulate IL-1β and IL-6 release and induce cytotoxicity, particularly when environmentally aged and coated with adsorbed pollutants (Bishop et al., 2025).

Therapeutic strategies targeting the gut microbiome—including probiotics, prebiotics, and dietary interventions—have shown potential to restore microbial balance and alleviate gastrointestinal symptoms in PD (Yadav and Raj, 2025). However, their effects on disease progression remain limited, highlighting the need for a more comprehensive understanding of gut–immune–brain interactions (Tansey et al., 2022; Tan et al., 2021; Jin et al., 2024).

It should be noted that most studies reporting these effects have utilized pristine polystyrene nanoparticles at concentrations (typically 10–100 μg/mL) that exceed estimated environmental exposure levels by several orders of magnitude (Hernandez et al., 2025). Whether similar effects occur with environmentally aged particles or at chronic low doses remains to be established. Although the immunotoxic effects described derive primarily from *in vitro* or animal studies using acute, high-dose exposures that may not reflect chronic, low-dose environmental conditions, these findings collectively suggest that MNPs can disrupt immune homeostasis at multiple levels, from cellular stress responses and immune sensing to systemic immune imbalance, thereby creating a permissive environment for neuroinflammatory and neurodegenerative processes. Where available, we note discrepancies between experimental and estimated environmental concentrations.

## 5 Convergence of MNPs and neuroimmunity in PD: a hypothetical gut–brain axis framework

Emerging evidence supports a multi-level convergence between environmental plastic exposure and neuroimmune dysregulation in PD (Lin et al., 2026; Casella and Ballaz, 2024; Zhu et al., 2025; Muhammad et al., 2026; Liang X. et al., 2025; Liang et al., 2022; Jeong et al., 2024). Rather than acting as isolated toxic agents, MNPs appear to intersect with key pathogenic pathways—including innate immune activation, adaptive immune imbalance, mitochondrial dysfunction, and *α*-synuclein pathology—across peripheral and central compartments (Mans et al., 2025). PD may be particularly susceptible to chronic MNP exposure because several core pathogenic mechanisms in PD—including mitochondrial vulnerability, oxidative stress amplification, gut dysbiosis, and persistent microglial priming—closely overlap with the dominant biological effects induced by MNPs.

**Figure 2:**
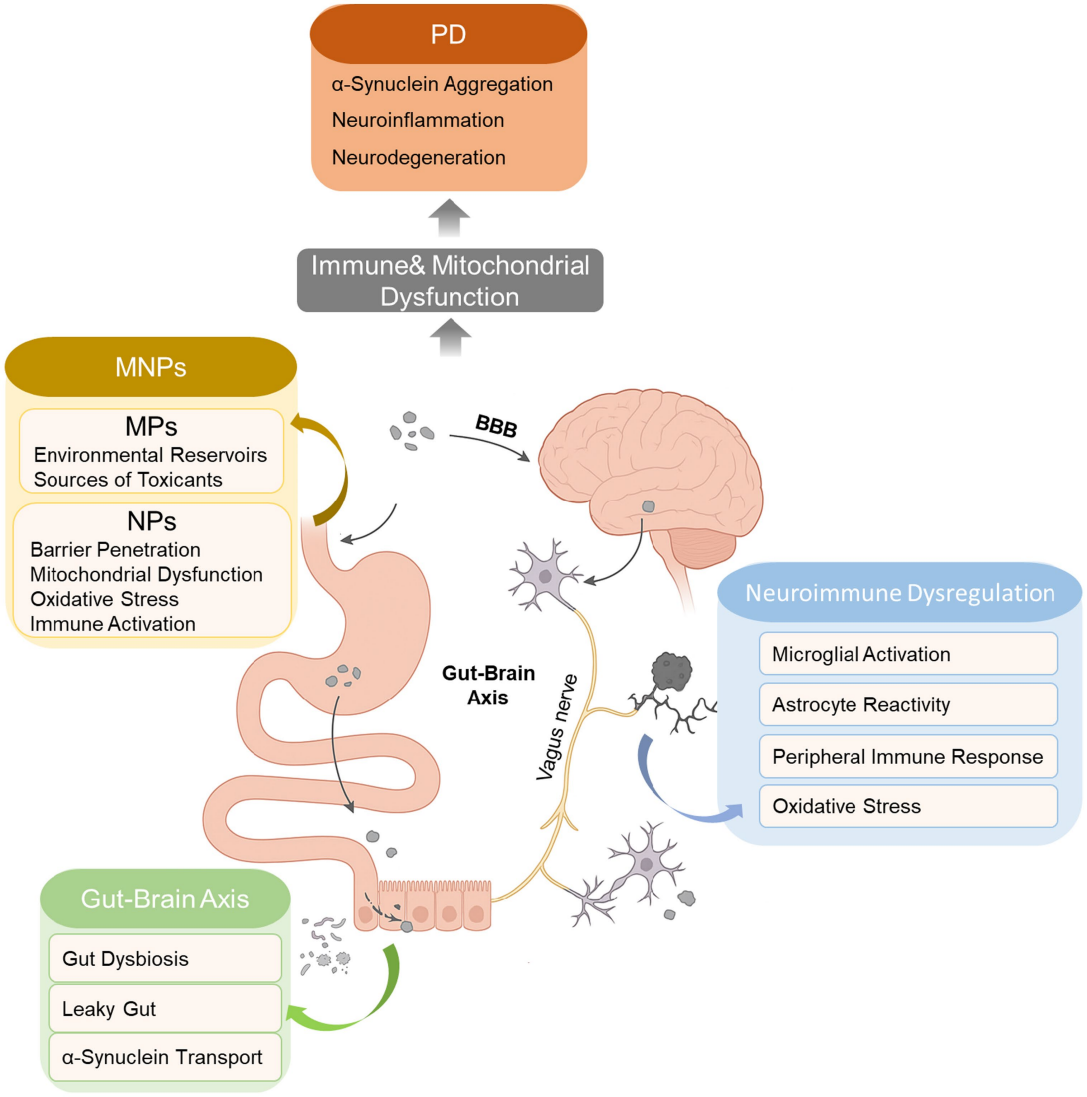
Convergence of micro- and nanoplastics with neuroimmune pathways in Parkinson’s disease. Schematic of a gut–brain model linking micro- and nanoplastics (MNPs) may modulate Parkinson’s disease (PD) pathogenesis. MNPs disrupt intestinal barrier integrity and microbiota composition, promoting enteric α-synuclein aggregation and systemic immune activation. Misfolded α-synuclein propagates to the brain via the vagus nerve. In parallel, NPs enter circulation, cross the blood–brain barrier (BBB), and activate microglia, inducing oxidative stress and inflammatory signaling. These peripheral and central pathways converge to amplify neuroinflammation and dopaminergic neuron degeneration, positioning MNPs as environmental amplifiers of PD pathogenesis.

We propose a conceptual model as a hypothetical framework to guide future research, in which MNPs function as environmental amplifiers within a gut–brain axis. A central feature of this model (Figure 2) is this axis as an early and permissive interface for MNP-induced effects. Ingestion of MNPs may alter gut microbial composition, disrupt intestinal barrier integrity, and promote systemic immune activation. This “leaky gut” state can facilitate the translocation of microbial products and inflammatory mediators into circulation, priming peripheral immune cells and lowering the threshold for neuroinflammatory responses. Simultaneously, MNP-induced dysbiosis may promote α-synuclein aggregation within the enteric nervous system, which can propagate to the brain via the vagus nerve, thereby linking environmental exposure to central neurodegeneration.

At the central level, experimental systems indicate that NPs can directly interact with microglia and astrocytes. Internalization of nanoscale particles induces lysosomal damage, mitochondrial dysfunction, and oxidative stress, leading to activation of the NLRP3 inflammasome and NF-κB signaling pathways. These events promote a shift toward pro-inflammatory microglial states, including DAM, which in turn drive astrocytic A1 polarization and amplify neuroinflammation. This glial crosstalk reinforces α-synuclein aggregation and dopaminergic neuron vulnerability (Paing et al., 2024; Kiran et al., 2025; Miyazaki and Asanuma, 2020).

Peripheral immune dysregulation further contributes to this convergence. MNP exposure might promote Th17-skewed responses and impair regulatory T-cell function, thereby compromising immune tolerance and facilitating chronic inflammation. These systemic alterations may enhance immune cell infiltration into the central nervous system or modulate microglial activation, establishing a bidirectional feedback loop between peripheral and central immunity (Greenland et al., 2025; Moquin-Beaudry et al., 2025).

Oxidative stress and mitochondrial dysfunction represent a shared mechanistic intersection across these pathways. In PD, impaired mitophagy and excessive reactive oxygen species (ROS) production promote α-synuclein misfolding and inflammatory signaling (Chang and Chen, 2020; Borsche et al., 2021; Wu et al., 2024; Kadac-Czapska et al., 2024). MNP-induced cellular stress may exacerbate these processes, reinforcing a feed-forward cycle of mitochondrial damage, immune activation, and neurodegeneration (Borsche et al., 2021; Zaman et al., 2021; Henrich et al., 2023).

Finally, MPs alter gut microbiota composition and intestinal immune tone (Sofield et al., 2024). Given the established role of the gut–brain axis in PD, microbiome disruption may represent an additional pathway through which environmental plastic exposure converges with neuroimmune dysfunction.

This convergence model positions MNPs not as primary etiological drivers, but as modulators that lower the threshold for neuroimmune activation in susceptible individuals. By integrating environmental exposure with gut, immune, and neural pathways, this framework provides a unifying perspective on how plastic pollution may contribute to PD pathogenesis.

## 6 Limitations, clinical implications and future directions

Several recent reviews have addressed MNP neurotoxicity, neuroinflammation, and gut dysbiosis in the context of neurodegenerative diseases. However, most existing syntheses examine these topics broadly across multiple disorders or focus on general neurotoxic mechanisms. The present review is specifically focused on PD-relevant neuroimmune pathways, with three distinctive features: (1) a detailed integration of central (microglial/astrocytic), peripheral (T cell, monocyte, NK cell), and enteric immune compartments within a unified PD framework; (2) a critical examination of the size-dependent paradigm distinguishing MPs (exposure burden) from NPs (biologically active fraction); and (3) a conceptual model positioning MNPs as environmental amplifiers within the gut–brain axis rather than primary etiological drivers. Where prior reviews have provided broad overviews of MNP neurotoxicity, we aim to offer a mechanistic, PD-specific synthesis that identifies specific immune nodes vulnerable to MNP modulation.

Despite growing mechanistic evidence, several critical challenges limit our current understanding of the relationship between MNP exposure and PD. First, the lack of standardized methodologies for detecting and quantifying MNPs in human tissues hampers reproducibility and cross-study comparisons. Variability in sampling, analytical techniques, and reporting metrics remains a major barrier to establishing reliable exposure–response relationships.

Second, most experimental studies rely on acute or high-dose exposures (typically 10–100 μg/mL *in vitro* or 0.1–10 mg/kg *in vivo*) that may not accurately reflect chronic, low-dose environmental conditions. Moreover, such high-dose studies cannot reliably inform risk assessment without dose–response data spanning environmentally relevant ranges. Given that PD develops over decades, understanding the long-term cumulative effects of MNP exposure is essential. In addition, current in vitro and animal models fail to fully capture the complex, multi-system interactions among the gut, immune system, and brain that characterize human disease. Reported organ accumulation frequently reflects transient detection rather than true long-term persistence, with limited distinction between parenchymal internalization and vascular sequestration.

Third, the heterogeneity of MNPs presents a significant challenge. Differences in particle size, polymer composition, surface chemistry, and associated contaminants complicate the attribution of biological effects to specific components. Most studies employ pristine, spherical, monodisperse polystyrene particles, whereas environmental MNPs are irregular in shape, polydisperse, chemically heterogeneous, and coated with environmental contaminants, microbial biofilms, and a protein corona that fundamentally alters their biological identity and toxicity. Contamination artifacts (e.g., airborne fibers, leaching from labware) are often inadequately controlled. Disentangling the relative contributions of MPs, NPs, and plastic-associated chemicals remains a key unresolved issue.

From a clinical and translational perspective, recognizing MNPs as potential environmental modifiers of neuroimmune vulnerability may expand current frameworks of PD risk. This perspective shifts the focus from genetic predisposition and classical toxins toward chronic, low-grade environmental exposures that interact with aging and immune dysregulation. To date, no prospective cohort studies or case–control studies have examined associations between measured MNP exposure and PD risk. This represents a critical gap, as the proposed model cannot be adequately tested without human data.

Future research should prioritize the following directions:

1) Longitudinal epidemiological studies: Large-scale, prospective cohorts are needed to evaluate associations between microplastic exposure and PD risk while controlling for demographic, genetic, occupational, and lifestyle confounders. Such studies would help identify susceptible populations and clarify dose–response relationships.
2) Mechanistic investigations: Further research should delineate the molecular pathways through which MPs and their associated additives influence neuroinflammation, oxidative stress, mitochondrial dysfunction, and *α*-synuclein aggregation. Particular emphasis should be placed on neuron–microglia–astrocyte interactions and systemic immune–brain crosstalk.
3) Standardized exposure assessment tools: Development of harmonized analytical methods, advanced imaging technologies, and validated biomarkers of internal microplastic burden is essential for improving exposure quantification and clinical translation.
4) Therapeutic and preventive strategies: A better understanding of how MPs amplify neuroinflammatory cascades may inform targeted interventions aimed at modulating immune and mitochondrial pathways in PD. Preventive measures—such as dietary modifications, reduction of plastic use, and improved air and water filtration systems—also warrant systematic evaluation as potential long-term risk mitigation strategies.

## 7 Conclusion

MNPs represent an emerging class of environmental factors with the potential to interact with key neuroimmune pathways implicated in PD. The role of MNPs in PD may be best understood not as direct toxicity but as environmental modifiers that integrate with aging, genetic susceptibility, and immune imbalance to shape disease vulnerability.

This review highlights two interconnected insights: first, a size-dependent paradigm in which MPs represent exposure burden while NPs drive biological effects; second, the gut–brain axis as a critical interface through which these particles may influence neurodegeneration.

By converging on shared pathways—including oxidative stress, mitochondrial dysfunction, innate and adaptive immune activation, and α-synuclein pathology—MNPs may lower the threshold for neuroinflammatory processes and accelerate disease progression in susceptible individuals. Although causality remains to be established, this integrative framework highlights the importance of considering chronic environmental exposures in models of neurodegenerative disease.

Understanding how MNPs interact with neuroimmune systems may not only refine our view of PD pathogenesis but also inform future strategies for prevention and intervention. More broadly, this perspective underscores a paradigm shift in which environmental micro-scale pollutants are recognized as active participants in complex, multi-system disease processes.
